# Health Disparities between the Rural and Urban Elderly in China: A Cross-Sectional Study

**DOI:** 10.3390/ijerph18158056

**Published:** 2021-07-29

**Authors:** Jian Zhang, Dan Li, Jianmin Gao

**Affiliations:** 1 School of Public Policy and Administration, Xi’an Jiaotong University, Xi’an 710049, China; zhjian.2004@stu.xjtu.edu.cn; 2 School of Public Management, Northwest University, Xi’an 710127, China; dandanli@nwu.edu.cn

**Keywords:** self-assessed health, disparity, rural, urban, elderly

## Abstract

Background: China is becoming an aging society, and the proportion of the population aged 60 years and above is increasing. There is a dualistic urban–rural economic structure between urban and rural areas in China, but there are few comparative health studies on the self-assessed health (SAH) status of the elderly between urban and rural areas. The aim of this study is to explore the SAH status of the elderly in China, and to identify the health disparity between the urban and rural elderly. Methods: The data from the fourth wave of the China Health and Retirement Longitudinal Study (CHARLS) in 2018 were adopted. A total of 9630 participants aged 60 and above were included in this study. SAH was used as the indicator, measuring the health status. Fairlie decomposition analysis was conducted to find the SAH disparity. Results: The proportion of good SAH of the rural elderly (24.01%) was significantly higher than the urban elderly (19.99%). The association of SAH was widely different between the rural and urban elderly. There was a stronger association between SAH and sleeping time in the urban elderly (Odds ratios (OR) = 3.347 of 4–8 h; OR = 3.337 of above 8 h) than the rural elderly (OR = 1.630 of 4–8 h; OR = 2.293 of above 8 h). Smoking and social activity were significant only in the urban elderly, while region and assets were significant only in the rural elderly. Drinking (11.45%), region (−33.92%), and assets (73.50%) were the main factors contributing to the urban–rural health disparities. Conclusions: This is the first comparative study examining SAH disparity, focusing on the elderly aged 60 and above in China. From the perspective of drinking, region, and assets, our study highlighted substantial urban–rural health disparities, and provided evidence for policy making on narrowing the health gap between urban and rural areas in China.

## 1. Background

Nowadays, people all around the world are experiencing increasing longevity and the total number of people aged 60 and above in the world is expected to increase from 900 million in 2015, to 2 billion in 2050. At the same time, China’s aging population is moving much faster than other developing countries [1]. By the end of 2020, the number of elderly people aged 60 and above in mainland China was 264 million, accounting for 18.7% of the total population. Since 2000, the proportion of the elderly population has increased by 8.4% [2]. It is predicted that by 2050, China’s population above 60 years of age may be reach 498 million [3]. With the marked decline in fertility, the increase in average life expectancy is leading to a rapid aging of the global population.

Human life extension will bring many benefits, but it relies heavily on the health of the elderly [4]. With the deepening of aging, more and more attention has been paid to the elderly’s health. Previous studies have discussed the difference between urban and rural residents’ health, however, there was no consensus on the issue. Fogelholm et al. [5] reported that the urban elderly had better physical health than the rural elderly, and most of the differences could be explained by educational background, physical activity, and smoking. Anderson et al. [6] used the 2013 County Health Rankings data to evaluate the differences between the rural and urban residents, and found that rural residents were more likely to have poorer health outcomes. They found that a variety of factors attributed to the differences, including the limitations in infrastructure, socioeconomic differences, insurance coverage deficiencies, and higher rates of traffic accidents. K.V. Smith and N. Goldman [7] used the Mexican Health and Aging Study to study the linkages of socioeconomic status (education, income, and wealth) and health, and found individuals with higher socioeconomic status were more likely to report better health than their lower counterparts in the urban areas.

The self-assessed health (SAH) was first proposed by Suchman et al. in 1958. They thought that SAH could comprehensively reflect the subjective feelings of the human body and objective physical functions, thereby obtaining a personal health status [8]. SAH is a subjective measure that integrates the biological, psychological, and social functions of the individual, and is also considered to be a sensitive measure of the overall health of the individual [9]. For example, Shadbolt et al. [10] found that SAH of the elderly aged 60 and above was valid, reliable, and responsive to change as a predictor of survival of advanced cancer. Jose et al. [11] thought that the SAH had been widely accepted as a reliable measure of overall health, since the concept of SAH was introduced in the early 20th century, meaning that many studies had proved the validity of SAH. Stina et al. [12] found that factors, such as diet quality, physical activity, alcohol consumption, smoking, and sleep duration influenced SAH. The empirical studies on the health and SAH of the elderly were carried out earlier, and the results had laid the foundation for the later in-depth study of health problems.

Because of the absence of the urban–rural division in foreign countries, there are few comparative studies on the health status of the elderly between urban and rural areas. However, due to China’s urban–rural dual structure, and the differences in the economic development between the urban and rural areas, as well as factors such as the living environment, the health difference between the urban and rural elderly is becoming increasingly serious in China [13,14]. For China, it is a hot research topic to discuss health issues, according to the urban and rural attributes of the elderly. Thus far, there have been many studies on the influencing factors of the elderly’s health in China, which have involved population characteristics, economic status, social capital, family support, living style, and life care; the results have provided inspiration and references for our research. For example, Zeng et al. [15] used data from the 2011 China National Survey of Aging Health and Longevity (CLHLS), and found that the rural elderly population reported better physical health than the urban elderly, and the urban elderly reported better mental health than the rural elderly. Li et al. [16] also used CLHLS data and found that there was a significant difference in the elderly’s health between urban and rural areas. Ren et al. [17] used 2010–2012 CHARLS data, and found income was a significant impact on the elderly’s health, and it was a greater impact on the rural elderly than the urban elderly. The urban–rural dual structure is an important influencing factor of the elderly’s health.

In terms of research methods, most studies that have focused on SAH of the urban and rural elderly and its influencing factors have done so through multiple regression methods; however, the method could not quantitatively explain the contribution of the differences between the two groups. Therefore, to further explore the factors leading to the difference in SAH between the urban and rural elderly, it is necessary to use decomposition technology. Interestingly, however, we know very little about the underlying causes of health disparity of the elderly by Fairlie decomposition. Should the dualistic urban–rural economy structure be viewed as a disparity that has important externalities, such as education, age, or job? Tao et al. [13] used Fairlie decomposition analysis, and found that 51.29% of the urban–rural disparity in the elderly population’s health could be explained by observable factors, and 48.61% by regional characteristics of the elderly’s residence. However, Tao et al. did not include the economic status in their analysis, and did not to mention the contribution of economic status to the urban–rural disparity. In addition, Xie et al. [18] used the Oaxaca decomposition and Fairlie decomposition to analyze the factors that affect the differences in health among three regions in China, but they only focused on the region disparity and ignored the differences in the urban and rural areas. Kan et al. [19] investigated the disparity and factors of SAH between male and female elderly people (aged 65 and above) by the Fairlie decomposition, and found that the SAH of the male elderly was better than that of the female elderly. Although there are several previous studies on the SAH disparity, we know very little about the underlying causes of these differences in terms of dualistic urban–rural economy structure.

Therefore, this study aimed to decompose the disparities in SAH between the rural and urban elderly in China into its contributory factors. This study may contribute to the literature on health of the elderly in China, and more importantly, the results also can provide reference for narrowing the health differences between urban and rural elderly.

## 2. Methods

### 2.1. Data

In this study, we used the fourth wave survey data from the China Health and Retirement Longitudinal Study (CHARLS), which was released in 2020. CHARLS is a set of high-quality micro-data, representing the households and individuals aged 45 and above in China. These data are used to analyze the problem of population aging in China and to promote interdisciplinary research on aging. To ensure the representativeness, the CHARLS baseline survey covered 150 countries/districts and 450 villages/urban communities across the country, involving 17,708 individuals in 10,257 households, reflecting the middle-aged and older Chinese population collectively. A stratified multi-stage probability proportionate to size sampling (PPS) and random sampling strategy was adopted [20]. The samples can be tracked every two to three years in the future, and one year after the survey, the data are open to the academic community. Ethical approval for all the CHARLS waves was granted from the Institutional Review Board (IRB) at Peking University. The IRB approval number for the main household survey, including anthropometrics, is IRB00001052-11015; the IRB approval number for biomarker collection is IRB00001052-11014. All the participants provided signed informed consent at the time of participation.

In 1956, the United Nations defined the elderly as 65 years and above in developed countries, and 60 years and above in developing countries [21]. By this standard, we defined the elderly as 60 years and above in China. In this study, after age screening (≥60 years old), 10,577 participants were selected. After data clean-up, excluding respondents of missing SAH (*n* = 906) and missing rural/urban sources (*n* = 53), 9630 respondents were finally selected. The rural elderly accounted for 75.52%, and the urban elderly accounted for 24.48%.

### 2.2. Self-Assessed Health Status Measurement

SAH is an indicator used to measure the health status of the elderly. In the CHARLS questionnaire, SAH can be reflected as the following question:

Question: How would you rate your health status? Would you say your health is very good, good, fair, poor, or very poor?

1. Very good
2. Good
3. Fair
4. Poor
5. Very poor

We defined “Very good”, and “Good” as 1, and “Fair”, “Poor”, and “Very poor” as 0. We set 1 = good SAH, 0 = bad SAH.

### 2.3. Description of Variables

According to Grossman’s theory of healthy production and related literature, the factors of the health status of residents can be divided into personal characteristics, economic situation, lifestyle, social support, and so on [22]. Numerous variables are available in CHARLS. Followed by prior empirical investigations [9,23,24,25,26], all possible variables that may produce SAH of the elderly were considered in our study. The definition and measurement of variables in this study were all shown in Table 1. 

### 2.4. Statistical Analysis

Firstly, the demographic characteristics of the elderly in urban and rural areas were described as frequencies and percentages. The chi-square test was used to compare the differences for SAH status, with categorical variables between the urban and rural elderly. Secondly, logit regression was used to calculate the odds ratios (OR) of the covariates, to identify the influencing factors of SAH status between the rural and urban elderly. Thirdly, Fairlie decomposition, by Fairlie and Bartus, was used to analyze the contribution of SAH disparities between the rural and urban elderly based on logit models. The main purpose of Fairlie decomposition was to split SAH disparities into two components, explained and unexplained. The first part is explained by group differences in the distribution of observable variables and often regarded as “endowment”. The second part reflects the unobserved heterogeneity between the cohorts [27,28]. We set binary logit regression models for SAH of the urban and rural elderly as:

(1) $$Y_{r}=F(X_{r}\beta_{r})$$

(2) $$Y_{u}=F(X_{u}\beta_{u})$$

Fairlie decomposition can be written as:

(3) $$\bar{Y_{u}}-\bar{Y_{r}}=\left[\sum_{i=1}^{N^{u}}\frac{F(X_{i}^{u}\beta^{u})}{N^{u}}-\sum_{i=1}^{N^{r}}\frac{F(X_{i}^{r}\beta^{u})}{N^{r}}\right]+\left[\sum_{i=1}^{N^{r}}\frac{F(X_{i}^{r}\beta^{u})}{N^{u}}-\sum_{i=1}^{N^{r}}\frac{F(X_{i}^{r}\beta^{r})}{N^{r}}\right]$$

$\bar{Y_{r}}$ and $\bar{Y_{u}}$ separately represented the mean value of SAH of urban and rural elderly, r and u separately represented rural and urban elderly. X represented independent variables, including gender, age, educational status, etc. β represented the coefficient. F represented the relation of logit regression equation. i represented the individual in the sample. $N^{r}$ and $N^{u}$ separately represented the sample size of urban and rural. All statistical analyses were performed using Stata 15.0.

## 3. Result

### 3.1. Characteristics of the Participants 

Table 2 shows the descriptive statistics analysis that compared the rural elderly with the urban elderly. Overall, 20.98% of the elderly had good SAH, while 79.02% of the elderly had bad SAH. In the rural areas, there were slightly less males (49.85%) than females (50.15%), and the situation in the urban areas was similar. In both areas, about half of the elderly were 65 to 74 years old, one third were 60 to 64 years old and one fifth were above 75 years old. The share of oldest age group was somewhat larger in the urban areas. In both areas, those that were married and living with a spouse accounted for about 75%. In total, 62.01% of the urban elderly had social activities, and 45.12% of the rural elderly had social activities. Other characteristics of the participants are all shown in Table 2.

In addition, we used chi-square test to examine the differences for categorical variables, and the results are shown in Table 2. The results indicated that there were significant differences in many characteristics between the two groups. Compared with the rural elderly, the urban elderly were more likely to have better SAH, a higher level of education, a higher probability of getting married and living with a spouse, a higher probability of sleeping 4–8 h, a lower probability of smoking, a higher probability of drinking, have more social activities and physical activities, be more in a middle region, and be more richer and richest in assets.

### 3.2. Comparison of Variable Distribution in Different SAH Status

Table 3 shows how the SAH status was distributed between the rural and urban elderly. There were major differences in good SAH and bad SAH. Drinking and physical activities were significant factors in the elderly with good SAH (*p* < 0.05), but not with bad health.

### 3.3. Associations between SAH Status and Its Determinants

Table 4 shows a variety of variables associated with SAH status, and its determinants through logit regression models. In the rural elderly, male (OR = 1.227), sleeping time 4–8 h (OR = 1.630) and above 8 h (OR = 2.293), and drinking (OR = 1.375) were associated with better SAH. Meanwhile, in the urban elderly, sleeping time 4–8 h (OR = 3.347) and above 8 h (OR = 3.337), and drinking (OR = 1.635) were associated with better SAH. In addition, age above 75 (OR = 0.721), smoking (OR = 0.642), and social activity (OR = 1.469) were significant only in the urban elderly, while middle (OR = 0.697) and west (OR = 0.696) region, richer (OR = 1.311), and richest (OR = 1.522) assets were associated only in the rural elderly.

### 3.4. Decomposition Analysis

This study aimed to parse out SAH disparities between the rural and urban elderly. The health disparity of the two groups was showed in Table 5. In total, 42.39% of the health disparity of SAH was enlightened by the factors considered, and 57.61% was caused by the factor of urban and rural areas. Our findings confirmed that drinking (11.45%), region (−33.92%), and assets quantiles (73.50%) were highly significant in the explanation of differences in SAH (*p* < 0.05).

## 4. Discussion

As far as we know, this study was the first large-scale comparative study to examine the SAH disparity, specifically focusing on the elderly (aged 60 and above) in China, the country with the largest population of elderly. This paper provided new empirical evidence on the health outcomes between rural and urban elderly. In addition, we used Fairlie decomposition to describe the contribution of each factor of urban–rural differences in SAH status of the elderly.

Our research revealed that there were significant differences in SAH between the urban and rural elderly. The proportion of good SAH of elderly was significantly lower in the urban areas (19.99%) than in the rural areas (24.01%). The results were similar to the study in the U.S. conducted by Anderson et al. [6], which reported rural residents were more likely to report poorer health outcomes compared with the urban residents. This might be because the urban elderly lived in cities with better health-care services, providing a buffer against health risks, which was conducive to their SAH [29]. Moreover, it might be related to the poor health cognition and poor working environment of the rural elderly [30]. 

We found that drinking and physical activities were significant factors in elderly with good SAH, but not with bad health (Table 3). The elderly with bad health would be more likely to stop drinking than the elderly with good SAH, due to health selectivity [15]. This finding was different from some studies [31], which showed there was no association between SAH and drinking In line with other studies [32,33], the elderly who engaged in regular physical activity were healthier than those who did not.

Our logit regression results (Table 4) showed that there were some major differences in factors associated with SAH in the rural and urban elderly. Firstly, there was a stronger association between SAH and sleeping time of the urban elderly (OR = 3.347 of 4–8 h; OR = 3.337 of above 8 h) than the rural elderly (OR = 1.630 of 4–8 h; OR = 2.293 of above 8 h). The rural elderly were more likely to have sleep disturbances, due to poor living conditions and less opportunity to receive health guidance [34,35,36]. Secondly, smoking was significant only in the urban elderly. Compared with the rural elderly, the urban elderly generally had a higher constant pension in China, and, thus, had a stronger purchasing power for smoking [37]. Social activity was also significant, only in the urban elderly. This might be due to the retirement of the urban elderly. The urban elderly are not accustomed to life after retirement, which might cause them to feel lonely, useless, and other problematic emotions. On the other hand, most of the rural elderly generally did not retire in China, and their social activities usually did not change. Thirdly, region and assets were associated with SAH, only in the rural elderly. Region and assets represented economic status, and a higher economic status could improve living conditions and healthcare [38]. Generally, the urban elderly had better living conditions and better medical insurance than the rural elderly.

By Fairlie decomposition (Table 5), we found that drinking (11.45%), region (−33.92%), and assets quantiles (73.50%) were the factors associated with urban–rural disparities, which indicated that drinking and economic status (region and assets) increased disparities between the rural and urban elderly. These were important findings, in order to address the overall research questions. In addition, it should be pointed out that some factors, such as smoking and drinking, were important factors associated with bad SAH and good SAH by single factor analysis (Table 3); however, only drinking had a contribution to the urban–rural SAH disparity. This was found because the Fairlie decomposition could calculate the contribution of the factors to the urban–rural SAH disparities by multiple regressions between the rural and urban elderly.

Based on the urban–rural SAH disparities, our results have strong policy implications. Firstly, as for drinking, we suggest that the government should strengthen smoking education programs, to promote the healthy lifestyle of the elderly. Secondly, as for the region, we need to pay attention to the design of a well-functioning regional layout [39], and the middle and western regions are proposed to be tilted in the government budget for the elderly. Thirdly, as for assets, the health assistance program for poverty alleviation should be strengthened, for example, by promoting free medical examinations for the poor elderly, strengthening financial support for public health, improving the health function of medical insurance, and so on [40]. At the same time, differences in SAH between the rural and urban elderly in China were not only found in differences in health determinants, but also perceived and actual social discrimination. However, reducing drinking, narrowing the region gap, and strengthening the health assistance program for poverty alleviation may not be sufficient. Indeed, only richer rural and urban elderly have more opportunities to enjoy a healthy life and receive better healthcare.

## 5. Limitations

Our study also had several limitations. Firstly, we used a cross-sectional survey, which might make it difficult to examine changes at different stages. Secondly, SAH might have subjective shortcomings, and could not objectively show health status. Finally, there were many factors associated with the SAH of the elderly, and we could not analyze other factors. Despite these limitations, our findings may provide an insight on the comparison of the SAH disparity between the rural elderly and the urban elderly in China. We will collect more data and analyze more factors in the follow-up study, in order to verify the rationality of our results.

## 6. Conclusions

This study focused on the urban–rural SAH disparities in China. The SAH of the urban elderly was clearly better than those in the rural areas. Factors including drinking, region, and assets, need to be addressed to reduce rural–urban health disparities in China. Our findings may help provide evidence for health policies and intervention strategies to reduce differences of health status between the urban and rural elderly in aging China. Our study contributes to the ongoing and expanding literature on health disparities from the perspective of the urban–rural dual structure in China.

## Figures and Tables

**Table 1:** Definition and measurement of variables.

Type of Variables	Name of Variables	Variable Assignment
Dependent variable	SAH	Bad SAH = 0; Good SAH = 1
Grouping variable	Location of residential address	Rural = 0; Urban = 1
Demographic and sociological characteristics	Gender	Female = 0; Male = 1
	Age	60–64 = 1; 65–74 = 2; ≥75 = 3
	Education level	Below primary school = 1; Primary school = 2; Middle school and above = 3
	Marital status	Married and live with spouse = 1; Married but not live with spouse = 2; Divorced and don’t live together as a couple anymore = 3; Widower = 4; Never married = 5
	Minorities	Han = 0; Ethnic minorities = 1
	Religious beliefs	No = 0; Yes = 1
	Sleeping time	≤4 h = 1; 4–8 h = 2; >8 h = 3
	Smoking	No = 0; Yes = 1
	Drinking	No = 0; Yes = 1
	Social activity	No = 0; Yes = 1
	Physical activity	No = 0; Yes = 1
Economic status	Region	East = 1; Middle = 2; West = 3
	Assets quantiles	Poorest = 1; Poorer = 2; Middle = 3; Richer = 4; Richest = 5

Note: SAH means Self-assessed of Health Status; h means hours.

**Table 2:** Descriptive statistics of variables.

Variable	Rural *N* (%)	Urban *N* (%)	Total	*p*-Value
SAH				<0.001
Bad+	5819 (80.01)	1791 (75.99)	7610 (79.02)	
Good	1454 (19.99)	566 (24.01)	2020 (20.98)	
Demographic and Sociological Characteristics				
Gender				0.219
Female+	3674 (50.16)	1225 (51.97)	4899 (50.87)	
Male	3599 (49.84)	1132 (48.03)	4731 (49.13)	
Age				0.147
60–64+	2386 (32.81)	778 (33.01)	3164 (32.86)	
65–74	3541 (48.69)	1104 (46.84)	4645 (48.23)	
≥75	1346 (18.50)	475 (20.15)	1821 (18.91)	
Education level				<0.001
Below primary school+	4452 (61.22)	668 (28.34)	5120 (53.17)	
Primary school	1580 (21.72)	512 (21.72)	2092 (21.72)	
Middle school and above	1241 (17.06)	1177 (49.94)	2418 (25.11)	
Marital status				<0.001
Married and live with spouse+	5510 (75.76)	1811 (76.84)	7321 (76.02)	
Married but not live with spouse	253 (3.48)	71 (3.01)	324 (3.36)	
Divorced and don’t live together as a couple anymore	70 (0.96)	46 (1.95)	116 (1.21)	
Widower	1385 (19.04)	422 (17.90)	1807 (18.77)	
Never married	55 (0.76)	7 (0.30)	62 (0.64)	
Minorities				0.278
Han+	6750 (92.81)	2203 (93.47)	8953 (92.97)	
Ethnic minorities	523 (7.19)	154 (6.53)	677 (7.03)	
Religious beliefs				0.514
No+	6479 (89.08)	2111 (89.56)	8590 (89.20)	
Yes	794 (10.92)	246 (10.44)	1040 (10.80)	
Life-Style and Health Behavior				
Sleeping time				<0.001
≤4 h+	1716 (23.61)	387 (16.43)	2103 (21.84)	
4–8 h	4670 (64.22)	1847 (78.40)	6517 (67.70)	
>8 h	885 (12.17)	122 (6.17)	1007 (10.46)	
Smoking				<0.01
No+	3966 (54.55)	1382 (58.68)	5348 (55.56)	
Yes	3305 (45.45)	973 (41.32)	4278 (45.46)	
Drinking				<0.01
No+	5028 (69.15)	1551 (65.86)		
Yes	2243 (30.85)	804 (34.14)		
Social activity				<0.001
No+	4063 (55.88)	895 (37.99)	4958 (51.50)	
Yes	3208 (45.12)	1461 (62.01)	4669 (49.50)	
Physical activity				<0.01
No+	4108 (56.50)	1253 (53.17)	5361 (55.69)	
Yes	3163 (43.50)	1103 (46.83)	4266 (44.31)	
Economic Status				
Region				<0.001
East+	2474 (34.31)	620 (27.42)	3094 (32.67)	
Middle	2409 (33.41)	979 (43.30)	3388 (35.77)	
West	2327 (32.28)	662 (29.28)	2989 (21.56)	
Assets quantiles				<0.001
Poorest+	1890 (26.02)	328 (13.94)	2218 (23.06)	
Poorer	1866 (25.68)	319 (13.56)	2185 (22.72)	
Middle	1457 (20.06)	433 (18.40)	1890 (19.65)	
Richer	1290 (17.76)	509 (21.63)	1799 (18.70)	
Richest	762 (10.48)	764 (32.47)	1526 (15.87)	

Note: SAH means Self-assessed of Health Status; Han means Han nationality; h means hours; + reference levels in the regressions; virtual variables for chi-square test; N means number; % means percent.

**Table 3:** Comparison of variable distribution in different SAH status.

Variable	Bad SAH			Good SAH		
	Rural *N* (%)	Urban *N* (%)	*p*-Value	Rural *N* (%)	Urban *N* (%)	*p*-Value
Demographic and sociological characteristics						
Gender			0.208			0.463
Female	3017 (51.85)	959 (53.55)		657 (45.19)	266 (47.00)	
Male	2802 (48.15)	832 (46.45)		797 (54.81)	300 (53.00)	
Age			0.088			0.694
60–64	1845 (31.71)	556 (31.04)		541 (37.21)	222 (39.23)	
65–74	2876 (49.42)	855 (47.74)		665 (45.74)	249 (43.99)	
≥75	1098 (18.87)	380 (21.22)		248 (17.06)	95 (16.78)	
Education level			<0.001			<0.001
Below primary school	3567 (61.30)	517 (28.87)		885 (60.87)	151 (26.68)	
Primary school	1321 (22.70)	400 (22.33)		259 (17.81)	112 (19.79)	
Middle school and above	931 (16.00)	874 (48.80)		310 (21.32)	303 (53.53)	
Marital status			<0.01			0.138
Married and live with spouse	4398 (75.58)	1366 (76.27)		1112 (76.48)	445 (78.62)	
Married but not live with spouse	189 (3.25)	52 (2.90)		64 (4.40)	19 (3.36)	
Divorced and don’ t live together as a couple anymore	53 (0.91)	35 (1.95)		17 (1.17)	11 (1.94)	
Widower	1133 (19.47)	331 (18.48)		252 (17.33)	91 (16.08)	
Never married	53 (0.79)	7 (0.39)		9 (0.62)	0 (0.00)	
Minorities			0.269			0.795
Han	5401 (92.82)	1676 (93.58)		1349 (92.78)	527 (93.11)	
Ethnic minorities	418 (7.18)	115 (6.42)		105 (7.22)	39 (6.89)	
Religious beliefs			0.491			0.855
No	5191 (89.21)	1608 (89.78)		1288 (88.58)	503 (88.87)	
Yes	628 (10.79)	183 (10.22)		166 (11.42)	63 (11.13)	
Life-style and health behavior						
Sleeping time			<0.001			<0.001
≤4 h	1495 (25.70)	352 (19.66)		221 (15.21)	35 (6.18)	
4–8 h	3675 (63.17)	1347 (75.25)		995 (68.48)	500 (88.34)	
>8 h	648 (11.13)	91 (5.09)		237 (16.31)	31 (5.48)	
Smoking			<0.05			<0.05
No	3218 (55.31)	1048 (58.58)		748 (51.48)	334 (59.01)	
Yes	3354 (44.69)	741 (41.42)		705 (48.52)	232 (40.99)	
Drinking			0.134			<0.01
No	4127 (70.94)	1236 (69.09)		901 (62.01)	315 (55.65)	
Yes	2254 (29.06)	553 (30.91)		552 (37.99)	251 (44.35)	
Social activity			<0.001			<0.001
No	3297 (56.67)	728 (40.67)		766 (52.72)	167 (29.51)	
Yes	2521 (43.33)	1097 (59.33)		687 (47.28)	399 (70.49)	
Physical activity			0.144			<0.01
No	3322 (57.10)	987 (55.14)		786 (54.09)	266 (47.00)	
Yes	2496 (42.90)	974 (44.86)		667 (45.91)	300 (53.00)	
Economic status						
Region			<0.001			<0.001
East	1866 (32.32)	449 (26.11)		608 (42.34)	171 (31.62)	
Middle	1983 (34.34)	745 (43.31)		426 (29.67)	234 (43.25)	
West	1925 (33.34)	526 (30.58)		402 (27.99)	136 (25.14)	
Assets quantiles			<0.001			<0.001
Poorest	1578 (27.15)	259 (14.47)		312 (21.47)	69 (12.26)	
Poorer	1522 (26.19)	262 (14.64)		344 (23.68)	57 (10.12)	
Middle	1492 (19.91)	333 (18.60)		300 (20.65)	100 (17.76)	
Richer	997 (17.15)	390 (21.79)		293 (20.17)	119 (21.14)	
Richest	558 (9.60)	546 (30.50)		204 (14.55)	218 (38.72)	

Note: SAH means Self-assessed of Health Status; Han means Han nationality; h means hours; Virtual variables for Chi-square test; N means number; % means percent.

**Table 4:** Logit regression results of the SAH in the rural and urban elderly.

Variable	Rural			Urban		
	OR	[95% CI]		OR	[95% CI]	
Demographic and sociological characteristics						
Gender	1.227 *	1.019	1.478	1.472 *	1.088	1.991
Age						
65–74	0.851 *	0.744	0.974	0.733 **	0.584	0.920
≥75	0.867	0.720	1.043	0.721 *	0.531	0.979
Education level						
Primary school	0.679	0.577	0.799	0.819	0.605	1.108
Middle school and above	1.025	0.866	1.212	0.814	0.628	1.057
Marital status						
Married but not live with spouse	1.238	0.914	1.676	1.130	0.618	2.066
Divorced and don’ t live together as a couple anymore	1.207	0.654	2.226	0.706	0.317	1.573
Widower	1.057	0.895	1.246	1.133	0.848	1.516
Never married	0.715	0.345	1.481	1.000		
Minorities	1.078	0.849	1.368	1.197	0.796	1.799
Religious beliefs	1.085	0.898	1.310	1.126	0.804	1.577
Life-style and health behavior						
Sleeping time						
4–8 h	1.630 ***	1.386	1.919	3.347 ***	2.291	4.889
>8 h	2.293 ***	1.860	2.827	3.337 ***	1.893	5.882
Smoking	0.904	0.762	1.071	0.642 **	0.483	0.853
Drinking	1.375 ***	1.200	1.577	1.635 ***	1.299	2.059
Social activity	1.098	0.973	1.238	1.469 **	1.173	1.838
Physical activity	1.077	0.954	1.217	1.195	0.972	1.470
Economic status						
Region						
Middle	0.697 ***	0.605	0.804	0.931	0.733	1.182
West	0.696 ***	0.598	0.809	0.775	0.591	1.016
Assets quantiles						
Poorer	1.163	0.979	1.382	0.851	0.562	1.299
Middle	1.193	0.994	1.432	0.973	0.671	1.411
Richer	1.311 **	1.087	1.580	1.010	0.703	1.451
Richest	1.522 ***	1.230	1.885	1.224	0.869	1.723
Constant	0.154 ***	0.121	0.196	0.092 ***	0.053	0.158
Prob > chi2	<0.001			<0.001		
Number of observation	7202			2251		

Note: OR means odds ratios; CI means confidence interval; h means hours; * *p* < 0.05, ** *p* < 0.01, *** *p* < 0.001; all predictors entered the logit regression simultaneously.

**Table 5:** Fairlie decomposition of SAH disparity between rural and urban elderly.

Terms of Decomposition	SAH		
Difference	−0.0399		
Explained (%)	−0.0169 (42.39%)		
Explained			
Contribution to difference	Contribution (%)	[95% CI]	
Gender	4.01	−0.0018	0.0005
Age	2.14	−0.0009	0.0002
Education level	21.05	−0.0134	0.0063
Marital status	1.26	−0.0016	0.0012
Minorities	−0.32	−0.0002	0.0003
Religious beliefs	−0.70	−0.0002	0.0005
Sleeping time	−0.29	−0.0029	0.0030
Smoking	3.50	−0.0015	0.0003
Drinking	11.45 ***	−0.0028	−0.0011
Social activity	15.68	−0.0061	0.0008
Physical activity	2.65	−0.0012	0.0003
Region	−33.92 ***	0.0033	0.0082
Assets quantiles	73.50 *	−0.0198	−0.0051

Note: SAH means Self-assessed of Health Status; CI means confidence interval; * *p* < 0.05, *** *p* < 0.001.

## Data Availability

All the data we used have been publicly released on the CHARLS website: http://charls.pku.edu.cn/pages/data/2018-charls-wave4/zh-cn.html (accessed on 15 April 2021).

## Abbreviations

CHARLS	China Health and Retirement Longitudinal Study
SAH	Self-assessed of Health Status
OR	Odds ratios
CLHLS	China National Survey of Aging Health and longevity
IRB	Institutional Review Board
